# 3,5-Dimethyl-2,6-di­phenyl­piperidine

**DOI:** 10.1107/S160053681400470X

**Published:** 2014-03-08

**Authors:** S. Sathya, K. Prathebha, G. Usha, S. Abdul Basheer, S. Ponnuswamy

**Affiliations:** a PG and Research Department of Physics, Queen Mary’s College, Chennai-4, Tamilnadu, India; b PG and Research Department of Chemistry, Government Arts College, Coimbatore 641 018, Tamilnadu, India

## Abstract

In the title compound, C_19_H_23_N, the piperidine ring has a chair conformation. The phenyl rings are inclined to one another by 52.76 (16)°. One of the methyl substituents on the piperidine ring is axial while the other is equatorial, like the phenyl rings. In the crystal, mol­ecules are linked *via* C—H⋯π inter­actions, forming zigzag chains along [001].

## Related literature   

For the biological activity of piperidine derivatives, see: Parthiban *et al.* (2005 ▶, 2009*a* ▶,*b* ▶, 2011 ▶); Aridoss *et al.* (2007 ▶). For related structures, see: Aravindhan *et al.* (2009 ▶); Sugumar *et al.* (2013 ▶). For ring puckering analysis, see: Cremer & Pople (1975 ▶); Nardelli (1983 ▶).
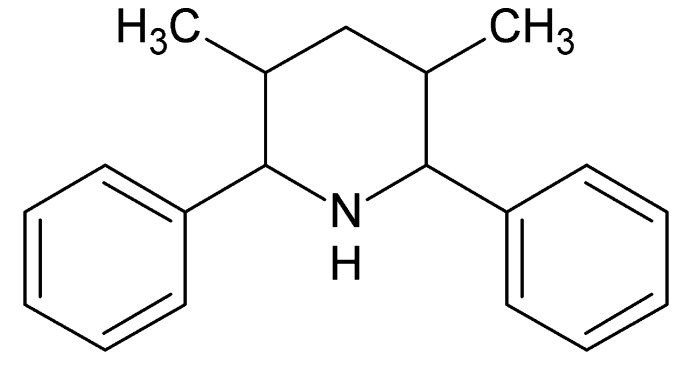


## Experimental   

### 

#### Crystal data  

C_19_H_23_N
*M* *_r_* = 265.38
Orthorhombic, 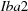

*a* = 10.1689 (8) Å
*b* = 43.141 (3) Å
*c* = 7.2658 (5) Å
*V* = 3187.5 (4) Å^3^
*Z* = 8
Mo *K*α radiation
μ = 0.06 mm^−1^
*T* = 293 K
0.35 × 0.30 × 0.25 mm

#### Data collection  

Bruker Kappa APEXII CCD diffractometer
Absorption correction: multi-scan (*SADABS*; Bruker, 2004 ▶) *T*_min_ = 0.978, *T*_max_ = 0.984
8658 measured reflections
3900 independent reflections
2652 reflections with *I* > 2σ(*I*)
*R*_int_ = 0.022

#### Refinement  

*R*[*F* ^2^ > 2σ(*F* ^2^)] = 0.051
*wR*(*F* ^2^) = 0.216
*S* = 0.78
3900 reflections
181 parameters
1 restraint
H-atom parameters constrained
Δρ_max_ = 0.22 e Å^−3^
Δρ_min_ = −0.24 e Å^−3^

### 

Data collection: *APEX2* (Bruker, 2004 ▶); cell refinement: *APEX2* and *SAINT* (Bruker, 2004 ▶); data reduction: *SAINT* and *XPREP* (Bruker, 2004 ▶); program(s) used to solve structure: *SIR92* (Altomare *et al.*, 1993 ▶); program(s) used to refine structure: *SHELXL97* (Sheldrick, 2008 ▶); molecular graphics: *ORTEP-3 for Windows* (Farrugia, 2012 ▶) and *Mercury* (Macrae *et al.*, 2008 ▶); software used to prepare material for publication: *SHELXL97*.

## Supplementary Material

Crystal structure: contains datablock(s) I, New_Global_Publ_Block. DOI: 10.1107/S160053681400470X/su2706sup1.cif

Structure factors: contains datablock(s) I. DOI: 10.1107/S160053681400470X/su2706Isup2.hkl

Supporting information file. DOI: 10.1107/S160053681400470X/su2706Isup3.cml

CCDC reference: 989359

Additional supporting information:  crystallographic information; 3D view; checkCIF report

## Figures and Tables

**Table 1:** Hydrogen-bond geometry (Å, °) *Cg*1 and *Cg*2 are the centroids of rings C1–C6 and C12–C17, respectively.

*D*—H⋯*A*	*D*—H	H⋯*A*	*D*⋯*A*	*D*—H⋯*A*
C5—H5⋯*Cg*1^i^	0.93	2.90	3.187 (2)	170
C18—H18*C*⋯*Cg*2^ii^	0.96	2.99	3.762 (5)	139

Symmetry codes: (i) 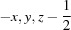; (ii) 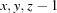.

**Fig. 1.**
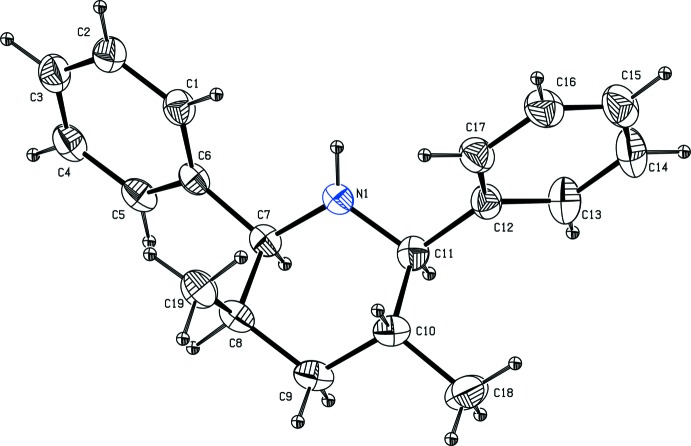
The molecular structure of the title molecule, with atom labelling. Displacement ellipsoids are drawn at the 30% probability level.

**Fig. 2.**
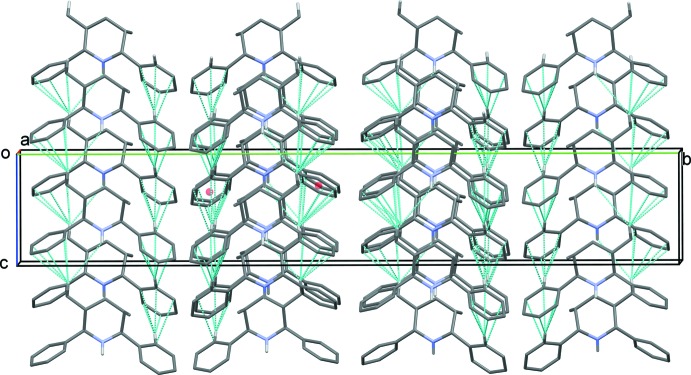
A view along the a axis of the crystal packing of the title compound. The centroids of phenyl rings C1—C6 and C12—C17 are marked with red dots and the dashed lines indicate the C—H···π interactions (see Table 1).

## supplementary crystallographic information

### 1. Comment

Piperidone molecules exhibit a broad-spectrum of biological activities ranging from anti-bacterial to anti-cancer (Parthiban *et al.*, 2005, 2009*b*, 2011). Isolation from natural products as well as synthesis of new molecules and the stereochemical analysis of this class of compounds are important in the field of medicinal chemistry. 2,6-disubstituted piperidones and their N-substituted derivatives are of great importance due to their significant pharmacological properties (Parthiban *et al.*, 2009*a*; Aridoss *et al.*, 2007).

The molecular structure of the title molecule is illustrated in Fig. 1. The bond distances and angles are normal and close to those observed previously for similar compounds (Aravindhan *et al.*, 2009; Sugumar *et al.*, 2013).

The piperidine ring adopts a chair conformation as defined by the puckering parameters (Cremer & Pople, 1975) and the smallest displacement asymmetry parameters (Nardelli, 1983) for the piperidine ring are q2 = 0. 0326 (3) Å, q3 = -0.579 (3) Å, QT = 0.5803 (3) Å,Theta2 _2_ = 176.78 (3)° and D2(C7—N1) = 0.0050 (1) Å. The dihedral angle between the two phenyl rings is 52.80 (2)°.

In the crystal, molecules are linked *via* C—H···π interactions forming zigzag chains along [001]; see Table 1 and Fig. 2.

### 2. Experimental

A mixture of 3,5-dimethyl-2,6-diphenylpiperidin-4-one (10 mmol) and 80% hydrazine hydrate (3.1 ml) in diethylene glycol (40 ml) was heated on a steam bath for 2 h. Potassium hydroxide pellets (2.8 g) were added to the mixture and the contents were refluxed vigorously on a heating mantle for another 2 h and then the reaction mixture was cooled. The product was filtered and recrystallized from ethanol yielding block-like colourless crystals.

### 3. Refinement

H atoms were positioned geometrically and treated as riding atoms: C—H = 0.93–0.98 Å, N—H =0.86 Å with *U*_iso_(H)= 1.5*U*_eq_(*C*-methyl) and = 1.2*U*_eq_(N,*C*) for other H atoms.

### Crystal data

**Table:**

C_19_H_23_N	*Z* = 8
*M**_r_* = 265.38	*F*(000) = 1152
Orthorhombic, *I**b**a*2	*D*_x_ = 1.106 Mg m^−^^3^
Hall symbol: I 2 -2 c	Mo *K*α radiation, λ = 0.71073 Å
*a* = 10.1689 (8) Å	µ = 0.06 mm^−^^1^
*b* = 43.141 (3) Å	*T* = 293 K
*c* = 7.2658 (5) Å	Block, colourless
*V* = 3187.5 (4) Å^3^	0.35 × 0.30 × 0.25 mm

### Data collection

**Table:**

Bruker Kappa APEXII CCD diffractometer	3900 independent reflections
Radiation source: fine-focus sealed tube	2652 reflections with *I* > 2σ(*I*)
Graphite monochromator	*R*_int_ = 0.022
ω and φ scan	θ_max_ = 28.3°, θ_min_ = 0.9°
Absorption correction: multi-scan (*SADABS*; Bruker, 2004)	*h* = −9→13
*T*_min_ = 0.978, *T*_max_ = 0.984	*k* = −57→45
8658 measured reflections	*l* = −9→9

### Refinement

**Table:**

Refinement on *F*^2^	Primary atom site location: structure-invariant direct methods
Least-squares matrix: full	Secondary atom site location: difference Fourier map
*R*[*F*^2^ > 2σ(*F*^2^)] = 0.051	Hydrogen site location: inferred from neighbouring sites
*wR*(*F*^2^) = 0.216	H-atom parameters constrained
*S* = 0.78	*w* = 1/[σ^2^(*F*_o_^2^) + (0.2*P*)^2^] where *P* = (*F*_o_^2^ + 2*F*_c_^2^)/3
3900 reflections	(Δ/σ)_max_ < 0.001
181 parameters	Δρ_max_ = 0.22 e Å^−^^3^
1 restraint	Δρ_min_ = −0.24 e Å^−^^3^

### Special details

**Table:**

Geometry. All e.s.d.'s (except the e.s.d. in the dihedral angle between two l.s. planes) are estimated using the full covariance matrix. The cell e.s.d.'s are taken into account individually in the estimation of e.s.d.'s in distances, angles and torsion angles; correlations between e.s.d.'s in cell parameters are only used when they are defined by crystal symmetry. An approximate (isotropic) treatment of cell e.s.d.'s is used for estimating e.s.d.'s involving l.s. planes.
Refinement. Refinement of *F*^2^ against ALL reflections. The weighted *R*-factor *wR* and goodness of fit *S* are based on *F*^2^, conventional *R*-factors *R* are based on *F*, with *F* set to zero for negative *F*^2^. The threshold expression of *F*^2^ > σ(*F*^2^) is used only for calculating *R*-factors(gt) *etc*. and is not relevant to the choice of reflections for refinement. *R*-factors based on *F*^2^ are statistically about twice as large as those based on *F*, and *R*- factors based on ALL data will be even larger.

### Fractional atomic coordinates and isotropic or equivalent isotropic displacement parameters (Å^2^)

**Table:**

	*x*	*y*	*z*	*U*_iso_*/*U*_eq_	
C1	0.2691 (3)	0.31401 (6)	0.3689 (4)	0.0682 (6)	
H1	0.3282	0.3302	0.3853	0.082*	
C2	0.2822 (3)	0.28735 (6)	0.4756 (5)	0.0776 (7)	
H2	0.3496	0.2857	0.5614	0.093*	
C3	0.1939 (3)	0.26338 (6)	0.4524 (5)	0.0800 (8)	
H3	0.2025	0.2453	0.5211	0.096*	
C4	0.0933 (3)	0.26627 (6)	0.3277 (4)	0.0763 (7)	
H4	0.0329	0.2503	0.3138	0.092*	
C5	0.0812 (2)	0.29289 (5)	0.2223 (3)	0.0661 (6)	
H5	0.0121	0.2946	0.1391	0.079*	
C6	0.1706 (2)	0.31695 (5)	0.2394 (3)	0.0562 (5)	
C7	0.1597 (2)	0.34446 (5)	0.1132 (3)	0.0573 (5)	
H7	0.0660	0.3479	0.0886	0.069*	
C8	0.2284 (2)	0.33925 (6)	−0.0733 (3)	0.0670 (6)	
H8	0.1847	0.3219	−0.1349	0.080*	
C9	0.2068 (3)	0.36835 (7)	−0.1906 (4)	0.0779 (7)	
H9A	0.2541	0.3660	−0.3058	0.093*	
H9B	0.1140	0.3701	−0.2194	0.093*	
C10	0.2518 (3)	0.39803 (6)	−0.0974 (4)	0.0702 (7)	
H10	0.3471	0.3968	−0.0789	0.084*	
C11	0.1862 (2)	0.40069 (5)	0.0914 (4)	0.0635 (6)	
H11	0.0910	0.4027	0.0734	0.076*	
C12	0.2343 (2)	0.42859 (5)	0.1987 (4)	0.0662 (6)	
C13	0.1532 (3)	0.45364 (6)	0.2290 (6)	0.0918 (10)	
H13	0.0681	0.4535	0.1821	0.110*	
C14	0.1971 (4)	0.47910 (6)	0.3290 (8)	0.1080 (13)	
H14	0.1413	0.4958	0.3498	0.130*	
C15	0.3227 (4)	0.47958 (7)	0.3968 (6)	0.1008 (12)	
H15	0.3521	0.4965	0.4651	0.121*	
C16	0.4041 (4)	0.45530 (6)	0.3639 (5)	0.0914 (9)	
H16	0.4902	0.4559	0.4070	0.110*	
C17	0.3604 (3)	0.42965 (7)	0.2667 (4)	0.0780 (7)	
H17	0.4168	0.4130	0.2474	0.094*	
C18	0.2231 (5)	0.42606 (9)	−0.2189 (6)	0.1090 (12)	
H18A	0.2522	0.4446	−0.1579	0.163*	
H18B	0.1303	0.4274	−0.2414	0.163*	
H18C	0.2688	0.4239	−0.3338	0.163*	
C19	0.3720 (3)	0.33137 (7)	−0.0545 (5)	0.0794 (8)	
H19A	0.4097	0.3285	−0.1745	0.119*	
H19B	0.3813	0.3126	0.0154	0.119*	
H19C	0.4167	0.3480	0.0075	0.119*	
N1	0.21151 (19)	0.37261 (4)	0.1973 (3)	0.0599 (5)	
H1A	0.2533	0.3727	0.3002	0.072*	

### Atomic displacement parameters (Å^2^)

**Table:**

	*U*^11^	*U*^22^	*U*^33^	*U*^12^	*U*^13^	*U*^23^
C1	0.0619 (13)	0.0621 (12)	0.0804 (17)	−0.0039 (9)	−0.0054 (12)	−0.0084 (12)
C2	0.0826 (18)	0.0710 (15)	0.0793 (18)	0.0082 (13)	−0.0061 (14)	−0.0033 (14)
C3	0.097 (2)	0.0625 (14)	0.0808 (18)	0.0029 (13)	0.0222 (16)	−0.0044 (13)
C4	0.0858 (17)	0.0669 (14)	0.0760 (16)	−0.0181 (12)	0.0179 (15)	−0.0179 (13)
C5	0.0602 (12)	0.0750 (13)	0.0630 (12)	−0.0131 (10)	0.0065 (11)	−0.0173 (11)
C6	0.0497 (10)	0.0601 (11)	0.0588 (11)	−0.0007 (8)	0.0057 (9)	−0.0152 (10)
C7	0.0464 (9)	0.0628 (11)	0.0628 (13)	−0.0018 (8)	−0.0011 (10)	−0.0086 (10)
C8	0.0657 (15)	0.0756 (14)	0.0598 (13)	−0.0068 (11)	0.0001 (11)	−0.0185 (11)
C9	0.0816 (16)	0.0959 (18)	0.0561 (13)	−0.0100 (14)	−0.0074 (12)	−0.0069 (13)
C10	0.0691 (14)	0.0829 (16)	0.0587 (14)	−0.0036 (12)	−0.0055 (11)	0.0032 (12)
C11	0.0549 (11)	0.0617 (13)	0.0740 (14)	0.0011 (9)	−0.0030 (12)	−0.0011 (11)
C12	0.0715 (14)	0.0552 (11)	0.0721 (15)	−0.0054 (10)	0.0068 (13)	−0.0006 (11)
C13	0.0821 (17)	0.0632 (14)	0.130 (3)	−0.0011 (12)	0.012 (2)	−0.0039 (17)
C14	0.126 (3)	0.0569 (15)	0.141 (3)	−0.0040 (16)	0.025 (3)	−0.0173 (19)
C15	0.132 (3)	0.0689 (18)	0.102 (2)	−0.0337 (18)	0.016 (2)	−0.0117 (17)
C16	0.099 (2)	0.0863 (18)	0.089 (2)	−0.0260 (16)	−0.0070 (18)	−0.0075 (15)
C17	0.0792 (16)	0.0765 (15)	0.0784 (17)	−0.0010 (13)	−0.0083 (14)	−0.0101 (13)
C18	0.138 (3)	0.107 (3)	0.081 (2)	−0.005 (2)	−0.019 (2)	0.026 (2)
C19	0.0694 (16)	0.0872 (17)	0.0817 (17)	0.0035 (13)	0.0160 (14)	−0.0152 (14)
N1	0.0650 (11)	0.0579 (10)	0.0569 (11)	−0.0023 (7)	−0.0026 (9)	−0.0064 (9)

### Geometric parameters (Å, º)

**Table:**

C1—C6	1.380 (4)	C10—H10	0.9800
C1—C2	1.394 (4)	C11—N1	1.458 (3)
C1—H1	0.9300	C11—C12	1.516 (4)
C2—C3	1.380 (4)	C11—H11	0.9800
C2—H2	0.9300	C12—C17	1.374 (4)
C3—C4	1.372 (5)	C12—C13	1.378 (4)
C3—H3	0.9300	C13—C14	1.391 (5)
C4—C5	1.386 (4)	C13—H13	0.9300
C4—H4	0.9300	C14—C15	1.369 (6)
C5—C6	1.385 (3)	C14—H14	0.9300
C5—H5	0.9300	C15—C16	1.356 (5)
C6—C7	1.504 (3)	C15—H15	0.9300
C7—N1	1.458 (3)	C16—C17	1.386 (4)
C7—C8	1.541 (3)	C16—H16	0.9300
C7—H7	0.9800	C17—H17	0.9300
C8—C19	1.506 (4)	C18—H18A	0.9600
C8—C9	1.533 (4)	C18—H18B	0.9600
C8—H8	0.9800	C18—H18C	0.9600
C9—C10	1.519 (4)	C19—H19A	0.9600
C9—H9A	0.9700	C19—H19B	0.9600
C9—H9B	0.9700	C19—H19C	0.9600
C10—C18	1.525 (4)	N1—H1A	0.8600
C10—C11	1.530 (4)		
			
C6—C1—C2	121.6 (2)	C11—C10—H10	108.2
C6—C1—H1	119.2	N1—C11—C12	109.3 (2)
C2—C1—H1	119.2	N1—C11—C10	109.5 (2)
C3—C2—C1	119.2 (3)	C12—C11—C10	112.3 (2)
C3—C2—H2	120.4	N1—C11—H11	108.5
C1—C2—H2	120.4	C12—C11—H11	108.5
C4—C3—C2	119.8 (3)	C10—C11—H11	108.5
C4—C3—H3	120.1	C17—C12—C13	118.4 (3)
C2—C3—H3	120.1	C17—C12—C11	120.8 (2)
C3—C4—C5	120.5 (2)	C13—C12—C11	120.8 (3)
C3—C4—H4	119.8	C12—C13—C14	120.7 (3)
C5—C4—H4	119.8	C12—C13—H13	119.7
C6—C5—C4	120.8 (2)	C14—C13—H13	119.7
C6—C5—H5	119.6	C15—C14—C13	120.0 (3)
C4—C5—H5	119.6	C15—C14—H14	120.0
C1—C6—C5	118.0 (2)	C13—C14—H14	120.0
C1—C6—C7	122.80 (19)	C16—C15—C14	119.6 (3)
C5—C6—C7	119.2 (2)	C16—C15—H15	120.2
N1—C7—C6	112.05 (18)	C14—C15—H15	120.2
N1—C7—C8	109.02 (19)	C15—C16—C17	120.7 (3)
C6—C7—C8	112.79 (19)	C15—C16—H16	119.6
N1—C7—H7	107.6	C17—C16—H16	119.6
C6—C7—H7	107.6	C12—C17—C16	120.6 (3)
C8—C7—H7	107.6	C12—C17—H17	119.7
C19—C8—C9	112.0 (2)	C16—C17—H17	119.7
C19—C8—C7	113.1 (2)	C10—C18—H18A	109.5
C9—C8—C7	107.7 (2)	C10—C18—H18B	109.5
C19—C8—H8	107.9	H18A—C18—H18B	109.5
C9—C8—H8	107.9	C10—C18—H18C	109.5
C7—C8—H8	107.9	H18A—C18—H18C	109.5
C10—C9—C8	113.5 (2)	H18B—C18—H18C	109.5
C10—C9—H9A	108.9	C8—C19—H19A	109.5
C8—C9—H9A	108.9	C8—C19—H19B	109.5
C10—C9—H9B	108.9	H19A—C19—H19B	109.5
C8—C9—H9B	108.9	C8—C19—H19C	109.5
H9A—C9—H9B	107.7	H19A—C19—H19C	109.5
C9—C10—C18	110.7 (3)	H19B—C19—H19C	109.5
C9—C10—C11	109.4 (2)	C11—N1—C7	114.0 (2)
C18—C10—C11	112.1 (3)	C11—N1—H1A	123.0
C9—C10—H10	108.2	C7—N1—H1A	123.0
C18—C10—H10	108.2		
			
C6—C1—C2—C3	−0.5 (4)	C9—C10—C11—N1	54.0 (3)
C1—C2—C3—C4	−1.2 (5)	C18—C10—C11—N1	177.1 (2)
C2—C3—C4—C5	1.2 (4)	C9—C10—C11—C12	175.7 (2)
C3—C4—C5—C6	0.6 (4)	C18—C10—C11—C12	−61.2 (3)
C2—C1—C6—C5	2.2 (4)	N1—C11—C12—C17	52.2 (3)
C2—C1—C6—C7	−175.6 (2)	C10—C11—C12—C17	−69.6 (3)
C4—C5—C6—C1	−2.3 (3)	N1—C11—C12—C13	−128.4 (3)
C4—C5—C6—C7	175.7 (2)	C10—C11—C12—C13	109.8 (3)
C1—C6—C7—N1	−30.0 (3)	C17—C12—C13—C14	−1.2 (5)
C5—C6—C7—N1	152.2 (2)	C11—C12—C13—C14	179.4 (3)
C1—C6—C7—C8	93.5 (3)	C12—C13—C14—C15	0.7 (6)
C5—C6—C7—C8	−84.3 (2)	C13—C14—C15—C16	0.8 (7)
N1—C7—C8—C19	67.8 (3)	C14—C15—C16—C17	−1.8 (6)
C6—C7—C8—C19	−57.4 (3)	C13—C12—C17—C16	0.2 (5)
N1—C7—C8—C9	−56.5 (2)	C11—C12—C17—C16	179.6 (3)
C6—C7—C8—C9	178.34 (19)	C15—C16—C17—C12	1.3 (5)
C19—C8—C9—C10	−69.9 (3)	C12—C11—N1—C7	175.44 (19)
C7—C8—C9—C10	55.1 (3)	C10—C11—N1—C7	−61.1 (3)
C8—C9—C10—C18	−178.0 (3)	C6—C7—N1—C11	−171.69 (18)
C8—C9—C10—C11	−54.1 (3)	C8—C7—N1—C11	62.7 (2)

### Hydrogen-bond geometry (Å, º)

Cg1 and Cg2 are the centroids of rings C1–C6 and C12–C17, respectively.

**Table:**

*D*—H···*A*	*D*—H	H···*A*	*D*···*A*	*D*—H···*A*
C5—H5···*Cg*1^i^	0.93	2.90	3.187 (2)	170
C18—H18*C*···*Cg*2^ii^	0.96	2.99	3.762 (5)	139

Symmetry codes: (i) −*x*, *y*, *z*−1/2; (ii) *x*, *y*, *z*−1.

## References

[bb1] Altomare, A., Cascarano, G., Giacovazzo, C. & Guagliardi, A. (1993). *J. Appl. Cryst.* **26**, 343–350.

[bb2] Aravindhan, S., Ponnuswamy, S., Jamesh, M., Ramesh, P. & Ponnuswamy, M. N. (2009). *Acta Cryst.* E**65**, o1974. 10.1107/S1600536809028049 PMC2977121 21583650

[bb3] Aridoss, G., Balasubramanian, S., Parthiban, P., Ramachandran, R. & Kabilan, S. (2007). *Med. Chem. Res.* **16**, 188–204.

[bb4] Bruker (2004). *APEX2*, *SAINT*, *XPREP* and *SADABS* Bruker AXS Inc., Madison, Wisconsin, USA.

[bb5] Cremer, D. & Pople, J. A. (1975). *J. Am. Chem. Soc.* **97**, 1354–1358.

[bb6] Farrugia, L. J. (2012). *J. Appl. Cryst.* **45**, 849–854.

[bb7] Macrae, C. F., Bruno, I. J., Chisholm, J. A., Edgington, P. R., McCabe, P., Pidcock, E., Rodriguez-Monge, L., Taylor, R., van de Streek, J. & Wood, P. A. (2008). *J. Appl. Cryst.* **41**, 466–470.

[bb8] Nardelli, M. (1983). *Acta Cryst.* C**39**, 1141–1142.

[bb9] Parthiban, P., Aridoss, G., Rathika, P., Ramkumar, V. & Kabilan, S. (2009*a*). *Bioorg. Med. Chem. Lett.* **19**, 2981–2985. 10.1016/j.bmcl.2009.04.038 19419867

[bb10] Parthiban, P., Balasubramanian, S., Aridoss, G. & Kabilan, S. (2005). *Med. Chem. Res.* **14**, 523–538.

[bb11] Parthiban, P., Balasubramanian, S., Aridoss, G. & Kabilan, S. (2009*b*). *Bioorg. Med. Chem. Lett.* **19**, 2981–2985. 10.1016/j.bmcl.2009.04.038 19419867

[bb12] Parthiban, P., Pallela, R., Kim, S. K., Park, D. H. & Jeong, Y. T. (2011). *Bioorg. Med. Chem. Lett.* **21**, 6678–6686. 10.1016/j.bmcl.2011.09.063 21983445

[bb13] Sheldrick, G. M. (2008). *Acta Cryst.* A**64**, 112–122. 10.1107/S0108767307043930 18156677

[bb14] Sugumar, P., Kayalvizhi, R., Mini, R., Ponnuswamy, S. & Ponnuswamy, M. N. (2013). *Acta Cryst.* E**69**, o609. 10.1107/S1600536813007927 PMC3629647 23634134

